# In Vivo Metabolite Formation and In Vitro Cytochrome P450-Mediated Metabolism of Vonoprazan in Horses

**DOI:** 10.3390/metabo16080584

**Published:** 2026-08-18

**Authors:** Camilo J. Morales, Daniel S. Mckemie, Jayanti Bhandari Neupane, Heather K. Knych

**Affiliations:** 1 K.L. Maddy Equine Analytical Chemistry Laboratory (Pharmacology Section), School of Veterinary Medicine, University of California, Davis, Davis, CA 95616, USA; cjaramillomorales@ucdavis.edu (C.J.M.); dsmckemie@ucdavis.edu (D.S.M.); jbhandarineupane@ucdavis.edu (J.B.N.); 2 Department of Molecular Biosciences, School of Veterinary Medicine, University of California, Davis, Davis, CA 95616, USA

**Keywords:** equine, gastric ulcers, metabolism, potassium-competitive acid blocker, CYP450, microsomes

## Abstract

**Background/Objectives:** Vonoprazan is a potassium-competitive acid blocker with potential for the treatment of equine gastric ulcer syndrome. Vonoprazan metabolic pathways in horses have not been characterized. This study aimed to identify vonoprazan metabolites following oral administration and to determine the metabolic enzymes responsible for their metabolism using in vitro models. **Methods:** Six healthy adult Thoroughbred horses received vonoprazan (0.5 and 1 mg/kg PO) in a randomized crossover design. Plasma concentrations of vonoprazan-N-oxide (M-I) and vonoprazan-nitrone (M-III) were quantified using a validated liquid chromatography-tandem mass spectrometry method, and a non-compartmental pharmacokinetic analysis was performed. In vitro metabolism was evaluated using equine liver microsomes (ELMs) and equine recombinant CYP450 (eq-rCYP) enzymes. Enzyme kinetics were characterized using nonlinear regression modeling. **Results:** Both metabolites were detected in plasma after oral administration. Systemic exposure to M-I was markedly greater than M-III at both doses. At 1 mg/kg, mean ± SD C_max_ values were 39.2 ± 28.3 ng/mL for M-I and 1.67 ± 1.66 ng/mL for M-III. AUC_0–∞_ increased dose-proportionally for both metabolites. In ELMs, M-I formation followed substrate inhibition kinetics, whereas M-III formation followed Michaelis–Menten kinetics. Among recombinant enzymes, CYP2D50 and CYP3A94 were the primary contributors to metabolite formation, exhibiting metabolite-specific kinetic profiles. **Conclusions:** These findings demonstrate that vonoprazan undergoes hepatic oxidative metabolism in horses, with M-I as the predominant circulating metabolite. Equine recombinants CYP2D50 and CYP3A94 appear to play central roles in equine vonoprazan metabolism, providing foundation for future evaluation of drug–drug interaction potential and clinical use in this species.

## 1. Introduction

Equine gastric ulcer syndrome (EGUS) has a high prevalence, ranging from 50 to 100% depending on the horse’s use. EGUS can impair athletic performance and adversely affect equine welfare, making it a condition of major importance to the equine industry worldwide [1,2]. Furthermore, approximately 77% of squamous disease cases and 50% or more of glandular disease cases are refractory to the current standard treatment with the proton pump inhibitor, omeprazole [3]. This supports the need for the development of alternative therapeutic agents.

Vonoprazan is a potassium-competitive acid blocker (P-CAB) with a mechanism of action related to, but distinct from, proton pump inhibitors, such as omeprazole. Vonoprazan inhibits the gastric H+/K+-ATPase by competitively blocking the potassium-binding site, thereby preventing the exchange of potassium for hydrogen ions and suppressing gastric hydrochloric acid secretion [4]. To date, only one study has described the pharmacokinetics of vonoprazan and its effect on gastric pH in horses. Results suggest promising therapeutic potential for the treatment of equine squamous and glandular gastric disease with this drug [5].

The metabolism of vonoprazan has been partially described in mice, dogs, and humans [6]. In clinical settings, vonoprazan has been associated with clinically significant drug–drug interactions related to metabolism. This includes attenuation of clopidogrel antiplatelet activity, increased drug concentrations following concurrent administration with narrow-therapeutic-index drugs such as tacrolimus, and reduced absorption of acid-dependent drugs due to potent gastric acid suppression [7,8,9]. Therefore, investigation of the metabolic pathways and identification of the enzymes involved in vonoprazan metabolism in horses is fundamental to better characterize its safety profile in this species.

In other species, vonoprazan is primarily metabolized in the liver by multiple cytochrome P450 (CYP) enzymes. The main CYP enzyme involved in vonoprazan metabolism in humans is CYP3A4; however, other enzymes, including CYP2B6, CYP2C9, CYP2C19, and CYP2D6, also contribute. The parent compound undergoes oxidative metabolism to generate multiple metabolites, including vonoprazan-N-oxide (M-I), M-II, and a non-oxidative derivate from M-I, vonoprazan-nitrone (M-III) [10]. These metabolites arise through parallel oxidative pathways rather than sequential metabolism [9]. In other species, these oxidative metabolites are considered pharmacologically inactive and do not contribute to gastric acid suppression [11]. In addition, in humans, vonoprazan undergoes sulfation to form M-IV, a reaction mediated by sulfotransferase SULT2A1; this metabolite is also inactive [9].

In the current study it was hypothesized that in horses, vonoprazan metabolism would be similar to that described in other species. To that end, the objectives of this study were (1) to determine if horses generate the M-I and M-III metabolites in vivo following oral administration of vonoprazan at doses of 0.5 and 1 mg/kg, and (2) to characterize the in vitro metabolism of vonoprazan, specifically to determine the enzymes responsible for vonoprazan metabolism in horses.

## 2. Materials and Methods

### 2.1. Metabolite Determination In Vivo

#### 2.1.1. Animals

Six university-owned Thoroughbred horses, including two mares and four geldings, aged between 7 and 10 years and weighing 449–660 kg, were enrolled in the study. All horses were deemed healthy based on physical examination and complete blood count. Horses were housed in 12 × 12-foot box stalls bedded with shavings. Horses were fed grass and alfalfa hay and had free access to water. No medications were administered for a minimum of 2 weeks prior to initiation of the study. The study protocol was approved by the Institutional Animal Care and Use Committee of the University of California, Davis (IACUC—protocol #23820).

#### 2.1.2. Drug Administration and Sample Collection

Drug administration and sample collection were conducted as described previously [5]. Briefly, vonoprazan was administered at doses of 0.5 and 1 mg/kg in a balanced crossover design. Blood samples were collected prior to and up to 72 h post drug administration and concentrations of vonoprazan and metabolites determined using liquid chromatography–tandem mass spectrometry (LC-MS/MS). Vonoprazan plasma concentrations have been reported previously [5].

#### 2.1.3. Determination of Vonoprazan Metabolite (M-I and M-III) Concentrations

Plasma calibrators were prepared by dilution of M-I and M-III working standard solutions with drug-free equine plasma to yield concentrations ranging from 0.1 to 300 ng/mL. Calibration curves and negative control samples were prepared fresh for each quantitative assay. Quality control samples consisted of equine plasma fortified with M-I and M-III at three concentrations (0.3, 10, and 80 ng/mL) and were included with each analytical batch.

Prior to analysis, 500 μL of acetonitrile (ACN):1 M acetic acid (9:1, *v*:*v*) containing 0.05 ng/μL of d3-vonoprazan (internal standard; Toronto Research Chemicals) was added to 500 μL of each sample, to precipitate proteins. Samples were vortexed for 2 min, refrigerated for 20 min, vortexed again for 1 min, and centrifuged at 4300 rpm (3830 *g*) for 10 min at 4 °C.

Plasma concentrations of the metabolites M-I and M-III were measured using a validated LC–MS/MS method operated in positive ion mode. Quantitative analysis was performed using a TSQ Altis triple quadrupole mass spectrometer coupled to a Vanquish liquid chromatography system (Thermo Scientific, San Jose, CA, USA). Chromatographic separation was achieved using an ACE 3 C18 column (10 cm × 2 mm; Mac-Mod Analytical) with a linear gradient of ACN in water, both containing 0.2% formic acid, at a flow rate of 0.35 mL/min. The initial ACN concentration was held at 5% for 0.5 min, increased to 90% over 5 min, and then returned to initial conditions for re-equilibration over 3 min.

Detection and quantification were performed using selective reaction monitoring (SRM) of the precursor ions for M-I (*m*/*z* 347), M-III (*m*/*z* 360), and the internal standard d3-vonoprazan (*m*/*z* 349). Corresponding product ions (M-I: *m*/*z* 205; M-III: *m*/*z* 68, 218, 313; d3-vonoprazan: *m*/*z* 173, 315) were monitored, and chromatographic peaks at the appropriate retention times were integrated using Quanbrowser software v4.1 (Thermo Scientific). Calibration curves for M-I and M-III were generated by linear regression with a weighting factor of 1/X. Linearity was confirmed with correlation coefficients ≥ 0.99 for both metabolites.

Assay precision and accuracy were assessed by analysis of quality control samples (n = 6/concentration) at low (0.3 ng/mL), medium (10 ng/mL), and high (80 ng/mL) concentrations for each metabolite. Accuracy, expressed as percent nominal concentration, for M-I was 4%, 2%, and 3% for 0.3, 10, and 80 ng/mL, respectively. Precision, expressed as percent relative standard deviation, was 106%, 92%, and 100% for 0.3, 10, and 80 ng/mL, respectively. For M-III accuracy was 93%, 101%, and 105% and precision was 6%, 2%, and 3% for the concentrations (ng/mL) 0.3, 10, and 80 respectively. The lower limit of quantification and limit of detection for M-I was 0.1 ng/mL and 0.05 ng/mL and for M-III was 0.1 ng/mL and 0.01 ng/mL, respectively.

#### 2.1.4. Identification of Glucuronide Conjugated Metabolites

To determine if vonoprazan metabolites (M-1 and M-III) were present as glucuronide conjugated metabolites in the blood following oral administration, the blood sample collected at 2 h post-administration from a single horse was subjected to additional analysis. Six aliquots were taken from the sample. Three aliquots were subjected to hydrolysis (B-glucuronidase 5000 U/mL overnight, room temp), and three were not hydrolyzed. All aliquots were subjected to extraction and analysis as described above. The concentration of M-I and M-III in hydrolyzed and non-hydrolyzed samples were compared. In addition, the non-hydrolyzed aliquots were subjected to full scan analysis from *m*/*z 475–575* to look for glucuronide conjugated metabolites.

#### 2.1.5. Pharmacokinetic Analysis

Non-compartmental pharmacokinetic analysis was performed for the metabolites M-I and M-III at both dose levels using commercially available software (Phoenix WinNonlin, version 8.3; Certara, Princeton, NJ, USA). The following parameters were calculated and reported: maximum plasma concentration (C_max_), time to maximum concentration (T_max_), terminal elimination rate constant (λ_z_), area under the plasma concentration–time curve extrapolated to infinity (AUC_0__→inf_), terminal elimination half-life (HL λ_z_), and the percentage of the total area under the curve extrapolated beyond the last measurable concentration (%AUC_extrap_).

### 2.2. In Vitro Study: Microsomal Metabolism and eq-CYP Enzyme Determination

#### 2.2.1. Chemicals

Tris-HCl, potassium chloride, potassium phosphate, formic acid, CHAPS (3-[(3-cholamidopropyl)-dimethylammonio]-propane-sulfonate), cytochrome c, and NADPH were obtained from MilliporeSigma (Burlington, MA, USA), vonoprazan fumarate used for enzyme incubations was obtained from Astral Scientific (Sydney, Australia), and cytochrome b5 was obtained from Thermo Fisher Scientific (Waltham, MA, USA).

#### 2.2.2. Microsomal Incubation with Vonoprazan

Equine liver microsomes (ELMs) were prepared from the liver of one healthy 3-year-old female Quarter Horse and stored at −80 °C, as previously described [12]. Upon thawing, microsomes were diluted to a protein concentration of 1 mg/mL in 100 mM potassium phosphate buffer and maintained on ice until use. Microsomal incubations were conducted in triplicate, with corresponding blank incubations (no NADPH). Prior to conducting the kinetic experiments, incubation conditions were optimized to ensure linearity with respect to protein concentration and incubation time. All subsequent incubations were performed within the linear range of the rate–concentration–time relationship. Microsomal reactions (final volume 500 μL) consisted of 1 mg/mL microsomal protein in 100 mM potassium phosphate buffer (pH 7.4), 1 mM CHAPS, and vonoprazan at varying concentrations (0–1000 μM). The total CYP450 activity in each incubation was 75 pmol. The mix was pre-incubated at 37 °C in a shaking water bath for 5 min prior to initiation of reactions by the addition of 2 mM NADPH. Reactions were allowed to proceed for 20 min and were terminated by the addition of 250 μL of ice-cold ACN.

#### 2.2.3. Incubations with cDNA-Expressed Cytochrome P450 Enzymes and Kinetic Assays

A library of equine recombinant cytochrome P450 (eq-rCYP) enzymes, previously generated by our laboratory, was used to determine the specific eq-rCYP isoforms involved in vonoprazan metabolism. This panel included 7 enzymes from the CYP2C family, 6 enzymes from the CYP3A family, 1 enzyme from the CYP2B family, 1 enzyme from the CYP4A family, 2 enzymes from the CYP2D family and 2 enzymes from the CYP1A family (Table 1). Incubations were performed using eq-rCYP enzymes in combination with NADPH cytochrome P450 oxidoreductase (POR). A ratio of 1:2 for eq-rCYP:POR and 1:4 for eq-rCYP:cytochrome b5 were used as previously described (12,13). Reaction mixtures containing 20 pmol of cytochrome b5, 10 pmol of POR, 100 mM potassium phosphate buffer (pH 7.4), 1 mM CHAPS, 2 mM NADPH, and 400 μM vonoprazan were pre-incubated for 5 min at 37 °C. Following pre-incubation 5 pmol of each eq-rCYP enzyme was added to the reaction for a final reaction volume of 250 μL. The incubation period was 30 min and was terminated by addition of 250 μL of ice-cold acetonitrile.

Subsequent to screening assays, full enzyme kinetic analyses were performed using the eq-rCYP isoforms demonstrating the greatest formation of both M-I and M-III during initial screening. For kinetic assays, the reaction mixtures (final volume 500 μL) contained NADPH cytochrome P450 oxidoreductase, recombinant cytochrome b5, 1 mM CHAPS, 2 mM NADPH, 100 mM potassium phosphate buffer (pH 7.4), and vonoprazan at concentrations ranging from 0 to 1000 μM. All incubations were performed under conditions verified to be within the linear range for enzyme activity and incubation time. Reactions were initiated by the addition of NADPH and terminated by addition of 250 μL of ice-cold ACN following an incubation period of 30 min [13].

#### 2.2.4. Determination of Metabolite Concentrations in Liver Microsomal and eq-rCYP Reaction

The M-I and M-III metabolites were quantified using LC–MS/MS as described above (Section 2.1.3).

#### 2.2.5. Kinetic Parameter Determination

For both microsomal and eq-rCYP450 incubations, metabolite concentrations were quantified for each individual replicate at each substrate concentration. Formation rates (velocity, V) were calculated independently for each replicate based on incubation time and conditions. Velocity was calculated using the formula:

(1) V=(concentration ∗ incubation volume)metabolite molecular weightincubation timeCYP450 activity

Kinetic parameters were determined through nonlinear regression analysis of individual velocity data using Graph Pad Prism software (GraphPad Software, v 9.0 San Diego, CA, USA). Data were fitted to single-site MM models to estimate V_max_ and K_m_**.** For reactions demonstrating decreased velocity at higher substrate concentrations consistent with substrate inhibition, data were fitted to a substrate inhibition model using the equation:

(2) Y= (Vmax∗ X)(Km+X) (1+XKi)

where K_i_ represents the substrate inhibition constant.

For reactions exhibiting sigmoidal behavior, an allosteric sigmoidal model equivalent to the Hill equation was applied using the equation:

(3) Y= (Vmax∗ Xh)(Khalf h+ Xh)

where K_half_ represents the substrate concentration producing half-maximal enzyme velocity, and h is the Hill coefficient reflecting curve steepness and providing an empirical indication of cooperativity.

When the Hill coefficient (h) equaled 1, the equation was considered equivalent to a single-enzyme MM model. When h exceeded 1 and the confidence interval excluded unity, the data were interpreted as demonstrating sigmoidal kinetics consistent with positive cooperativity. For reactions that did not achieve definable saturation within the tested concentration range and for which nonlinear models were unstable or statistically inadequate, apparent intrinsic clearance was estimated from linear regression of the initial concentration range.

For reactions described by the Michaelis–Menten model, intrinsic clearance (CL_int_) was calculated as V_max_/K_m_. For substrate inhibition models, intrinsic clearance was calculated as V_max_/K_m_ and reflects catalytic efficiency under low-substrate, non-inhibited conditions. For Hill kinetics, apparent intrinsic clearance was estimated from the initial slope of the fitted model as:

(4) =(Vmax∗ h)Khalf

The most appropriate kinetic model was selected based on the substrate concentration–velocity relationship and goodness-of-fit. Michaelis–Menten kinetics were used for hyperbolic saturation curves, substrate inhibition models for reactions showing decreased velocity at high substrate concentrations, and Hill kinetics for sigmoidal curves with a Hill coefficient significantly greater than 1 (95% confidence interval excluding 1). When saturation was not achieved or nonlinear models provided an inadequate fit, apparent intrinsic clearance was estimated by linear regression of the initial linear portion of the curve.

## 3. Results

### 3.1. In Vivo Study: Metabolite Determination in Plasma

All horses completed the study and tolerated the single oral administration of vonoprazan at both doses. Both metabolites (M-I and M-III) were detected at both doses. There was no difference in the concentration of either metabolite (M-I and M-III) between the samples subject to hydrolysis and those not subject to hydrolysis, suggesting the absence of glucuronide conjugated metabolites, at this time point. Pharmacokinetic parameters for the metabolites M-I and M-III derived from non-compartmental analysis are presented in Table 2.

**Figure 1:**
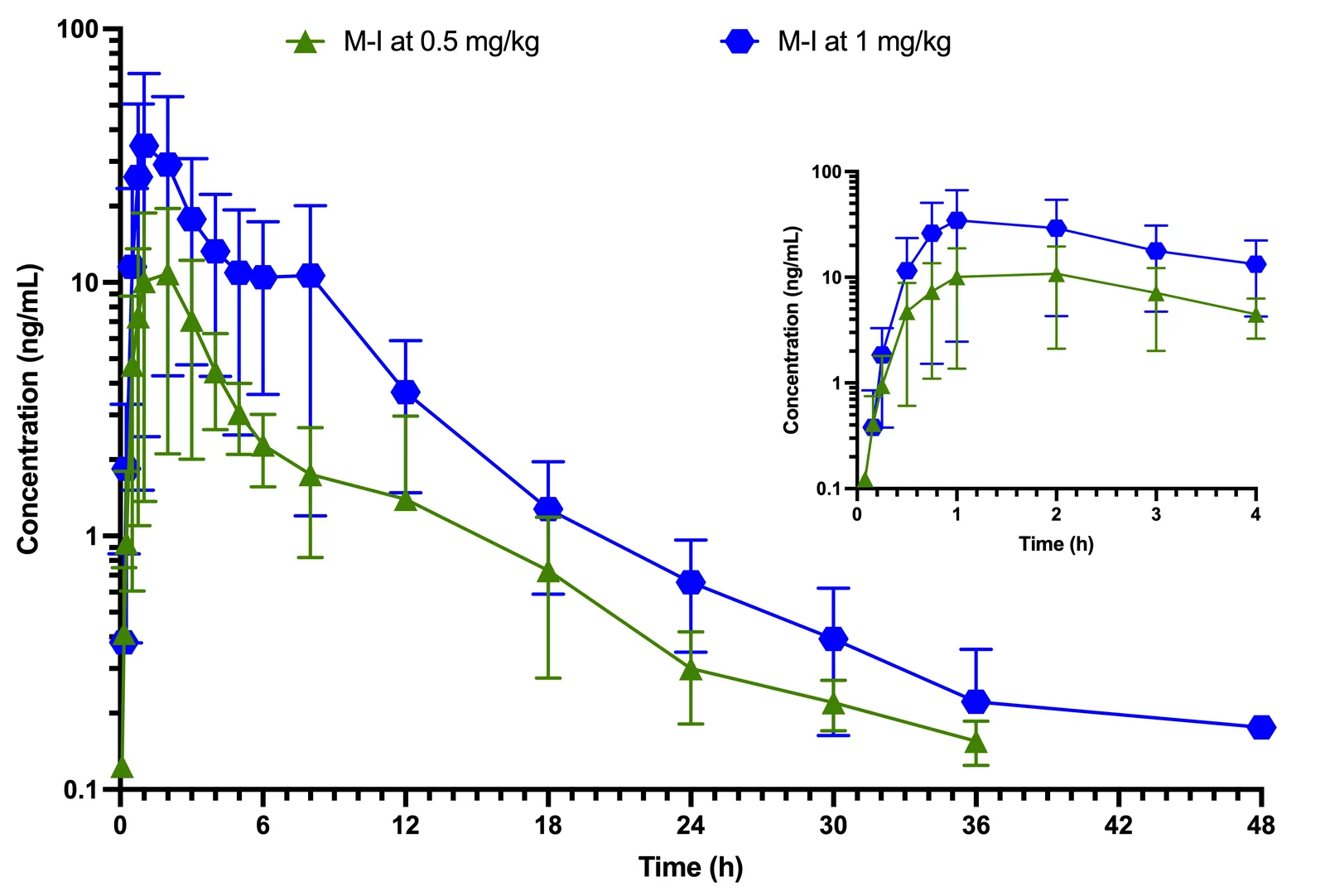
Plasma concentration–time profile of metabolite M-I following oral administration of vonoprazan at doses of 0.5 and 1 mg/kg. Data are presented as arithmetic mean ± standard deviation. The inset depicts the first 4 h post-administration.

**Figure 2:**
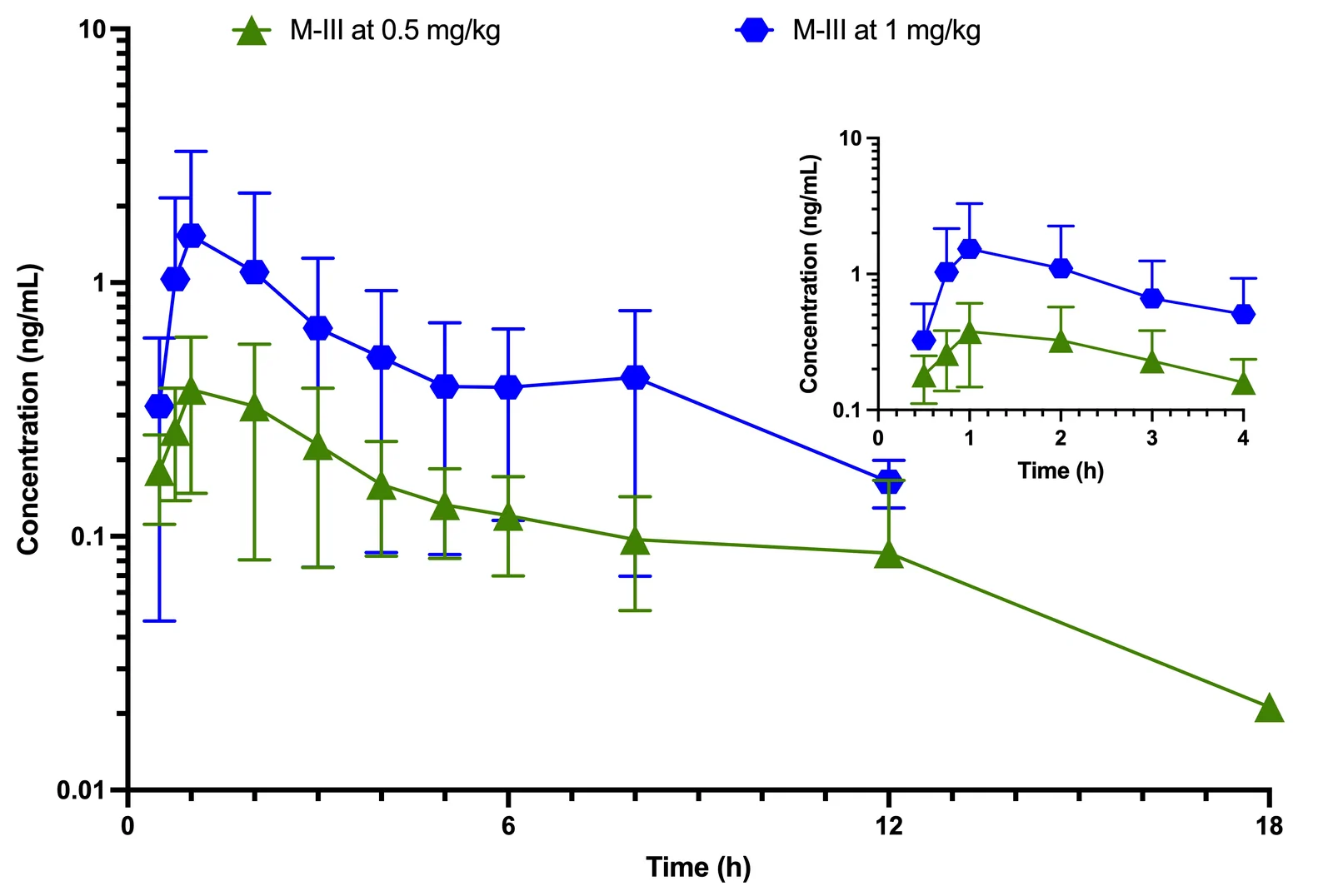
Plasma concentration–time profile of metabolite M-III following oral administration of vonoprazan at doses of 0.5 and 1 mg/kg. Data are presented as arithmetic mean ± standard deviation. The inset depicts the first 4 h post-administration.

Following oral administration of both doses of vonoprazan, plasma concentrations of the M-I metabolite were consistently greater than M-III (Figure 1 and Figure 2).

### 3.2. In Vitro Study: Microsomal Metabolism and eq-rCYP Enzyme Determination

**Figure 3:**
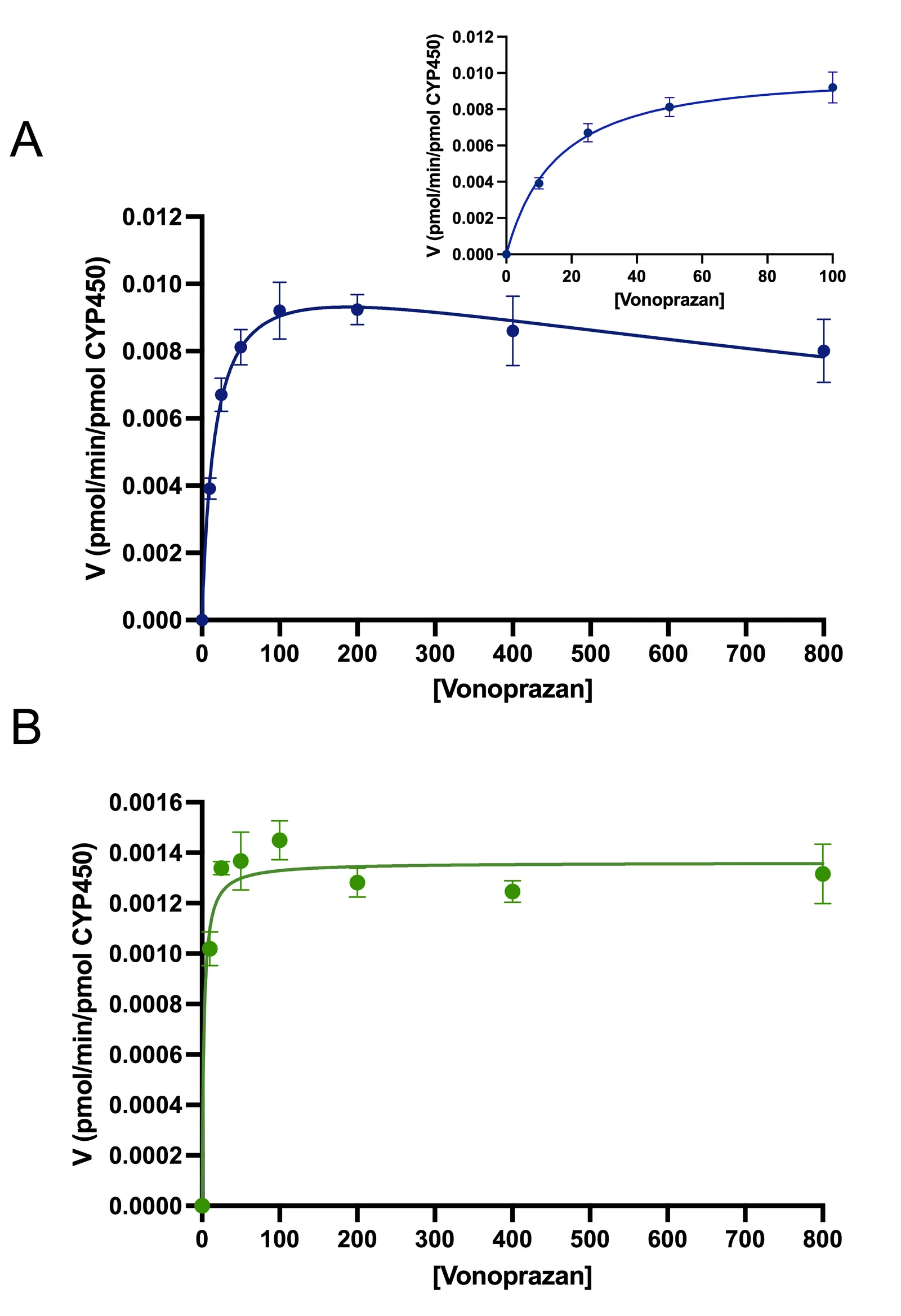
In vitro enzyme kinetics of vonoprazan metabolite formation in equine liver microsomes. (**A**) Formation of vonoprazan-N-oxide (M-I) in equine liver microsomes (ELMs). The inset highlights the initial concentration range. (**B**) Formation of vonoprazan-nitrone (M-III) ELMs. Data are presented as mean ± SD from triplicate incubations. Solid lines represent the best-fit nonlinear regression models for each system.

Equine liver microsomal incubations with vonoprazan generated the two metabolites (M-I and M-III) identified in the in vivo portion of the study. Formation of M-I deviated from classical Michaelis–Menten kinetics at higher substrate concentrations and was best fit by a substrate inhibition model (Figure 3A). In contrast, M-III formation followed classical Michaelis–Menten kinetics with no evidence of lack-of-fit (Figure 3B). Across the tested substrate concentrations, M-I exhibited a greater maximal velocity, whereas M-III demonstrated a lower apparent K_m_, consistent with higher substrate affinity under microsomal conditions. Kinetic parameters are summarized in Table 3.

**Figure 4:**
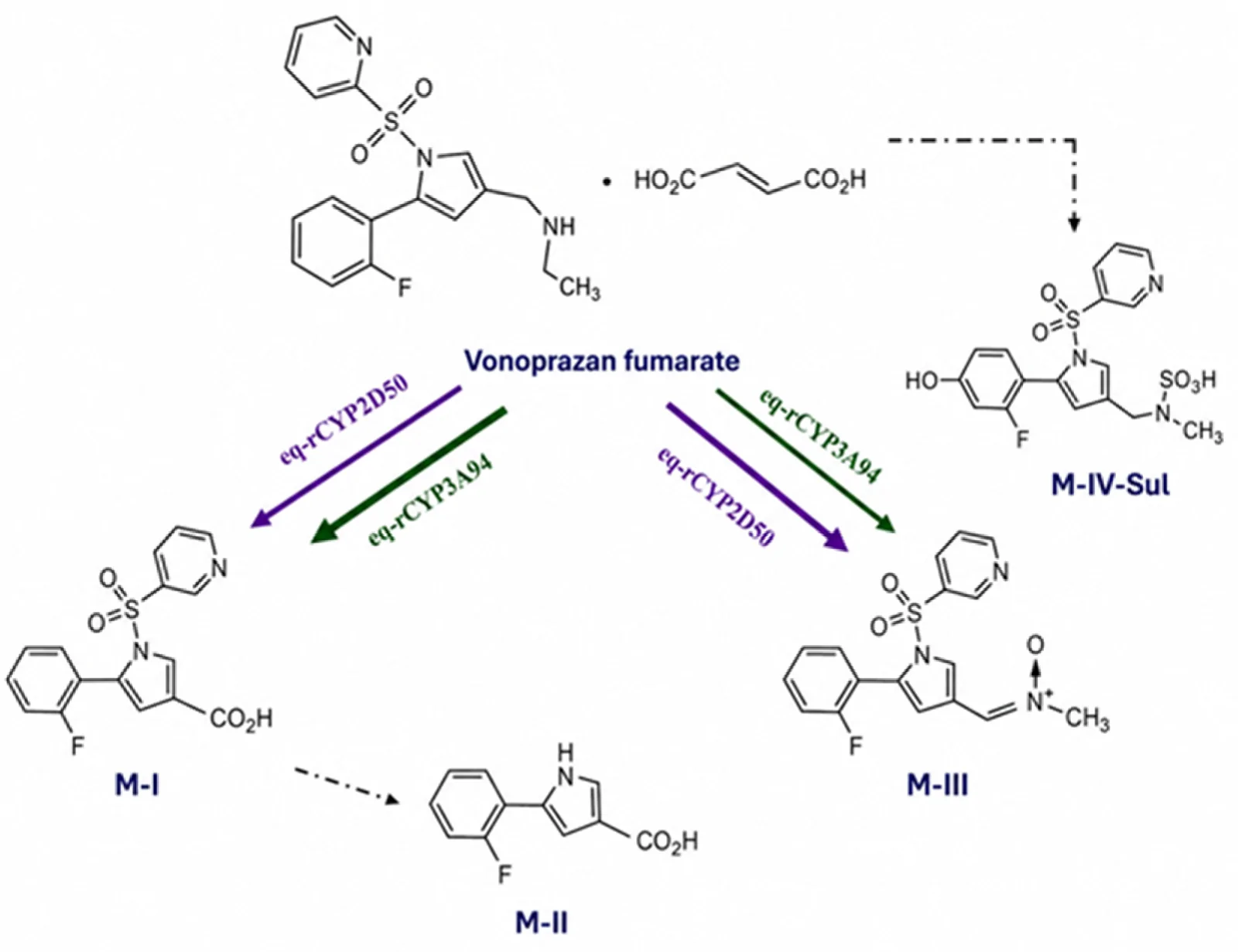
Proposed metabolic pathway for vonoprazan in horses. Vonoprazan is metabolized by the equine recombinant cytochrome P450 enzymes eq-rCYP2D50 and eq-rCYP3A94 to form the oxidative metabolites vonoprazan N-oxide (M-I) and vonoprazan nitrone (M-III). Dashed arrows indicate additional metabolites reported in other species (M-II and M-IV-Sul) that were not evaluated in the present study. Arrow thickness reflects the relative contribution of each recombinant CYP enzyme to metabolite formation, with thicker arrows indicating greater catalytic activity.

**Figure 5:**
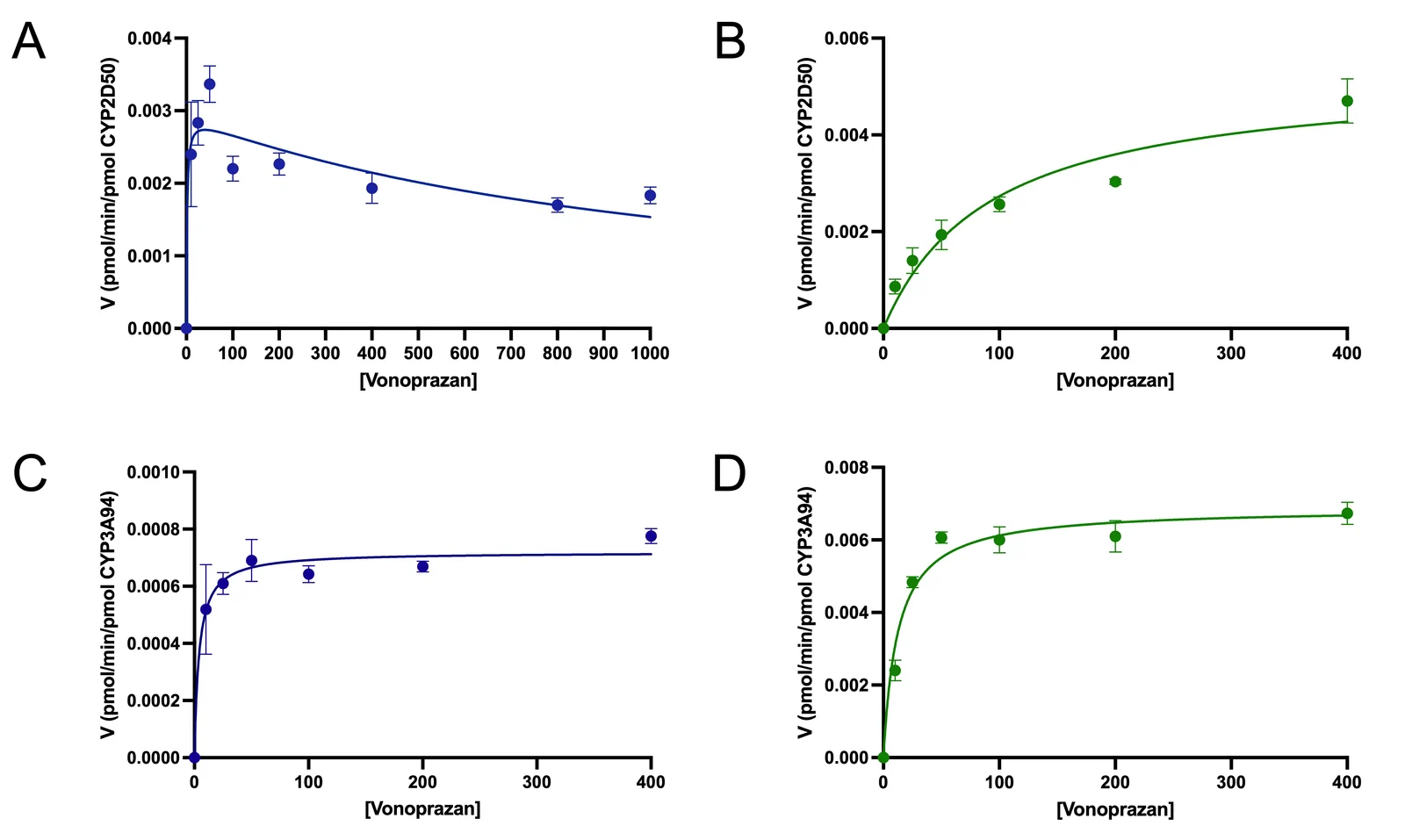
In vitro enzyme kinetics of vonoprazan metabolite formation in equine recombinant CYP isoforms. (**A**) Formation of M-I by recombinant CYP2D50. (**B**) Formation of M-III by recombinant CYP2D50. (**C**) Formation of M-I by recombinant CYP3A94. (**D**) Formation of M-III by recombinant CYP3A94. Data are presented as mean ± SD from triplicate incubations. Solid lines represent the best-fit nonlinear regression models for each system.

Screening of eq-rCYP450 enzymes identified eq-rCYP3A94 and eq-rCYP2D50 as the predominant enzymes responsible for M-III and M-I formation, respectively (Figure 4). These enzymes were selected for full enzyme kinetic studies. For eq-rCYP2D50, formation of M-I was fitted best by a substrate inhibition model (Figure 5A), whereas M-III formation did not reach saturation within the tested concentration range and increased linearly with substrate concentration. As a result, the initial linear portion of the curve was characterized by linear regression (Figure 5B).

For eq-rCYP3A94, M-I formation followed classical Michaelis–Menten kinetics (Figure 5C). In contrast, M-III formation was not sufficiently described by a single-site Michaelis–Menten model. Within the 0–400 µM concentration range, an allosteric sigmoidal (Hill) model provided the best fit and eliminated lack-of-fit. The Hill coefficient exceeded unity, consistent with positive cooperativity (Figure 5D). Kinetic parameters for all enzyme systems are summarized in Table 3.

## 4. Discussion

Vonoprazan administration to horses for the treatment of EGUS has been recently described [5]. The goal of the current study was to begin to characterize vonoprazan metabolism in horses, both in vivo and in vitro. Oral administration of vonoprazan to horses resulted in generation of the metabolites M-I and M-III. Concentrations for both metabolites were low, with M-I concentrations higher than M-III following administration of both doses. The terminal half-life was longer for M-I. At 1 mg/kg the AUC_0-inf_ for M-I was 180.1 ± 86.44 h*ng/mL. In comparison, reported AUC_0–24_ values for M-I in dogs and rats administered doses of 2 and 0.3 mg/kg were 312 ± 122 h*ng/mL and 4392 ± 51.9 h*ng/mL, respectively [14]. When accounting for dose differences and considering that the AUC calculated in the present study was extrapolated to infinity, which is greater than the AUC extrapolated over the first 24 h, these findings suggest that M-I formation in horses is more comparable to that observed in dogs than in rats.

In vitro findings were concordant with in vivo results in that vonoprazan metabolism in ELMs resulted in generation of low concentrations of both M-I and M-III, with M-I concentrations exceeding M-III across the tested concentration range. Similar to horses, M-I is reportedly the major circulating metabolite in humans [14,15], and its formation rate exceeds that of other vonoprazan metabolites in mice, rats, dogs, and monkey liver microsomes [15]. M-I formation was best described by a substrate inhibition model, consistent with inhibitory binding at higher substrate concentrations, a phenomenon commonly observed in CYP-mediated oxidations involving multiple substrate-binding interactions or conformational flexibility within the active site [16,17]. In contrast, M-III formation followed classic Michaelis–Menten kinetics in ELMs. Despite a lower apparent K_m_ for M-III, the substantially greater V_max_ and intrinsic clearance of M-I suggests that generation of M-I represents the dominant oxidative pathway in ELMs.

In the current study, eq-rCYP3A94 and eq-rCYP2D50 were the predominant enzymes associated with vonoprazan metabolism in horses. Other members of the CYP3A family (CYP3A89, CYP3A93, and CYP3A95) also contributed to vonoprazan biotransformation, primarily to the formation of M-III and M-I, but concentrations were much lower. These enzymes are equine orthologues of human CYP3A4, [18,19,20], which is the principal enzyme involved in vonoprazan metabolism in humans, rats, and beagle dogs [14]. CYP2D50 is regarded as the equine orthologue of human CYP2D6 [19] and was also associated with vonoprazan metabolism in the present study.

CYP2D50 exhibited higher affinity and catalytic efficiency for M-I formation and reproduced the substrate inhibition pattern observed in ELMs, supporting a principal role in M-I generation. By contrast CYP2D50-mediated M-III formation did not conform to any conventional enzymatic kinetic model. CYP3A94 displayed a different kinetic profile, with M-I formation following Michaelis–Menten kinetics and exhibiting lower intrinsic clearance than CYP2D50, whereas M-III formation demonstrated positive cooperativity, consistent with the atypical kinetic behavior previously described for CYP3A enzymes [21,22]. Collectively, these findings underscore enzyme- and metabolite-specific differences in affinity and catalytic efficiency, likely reflecting differential substrate orientation and binding dynamics within individual CYP isoforms.

The use of recombinant enzymes provided strong evidence of the role of CYP2D50 and CYP3A94 in vonoprazan metabolism in the horse. While recombinant CYP enzymes are commonly used for reaction phenotyping studies, it is important to note that complementary approaches such as chemical inhibition or immuno-inhibition strengthen identification of involvement of specific enzymes in metabolite generation [15]. Unfortunately, the lack of validated inhibitors of equine CYP enzymes and equine specific CYP inhibitor antibodies currently precludes confirmation using these methods in the current study.

Importantly, the in vitro findings were concordant with the in vivo plasma data. Following oral administration, M-I concentrations consistently exceeded those of M-III at both doses, mirroring the metabolic profile observed in ELMs. Similar to what was found in horses, M-I is reportedly the major circulating metabolite in humans [14,23], and its formation rate exceeds that of other vonoprazan metabolites in mice, rats, dogs, and monkey liver microsomes [23].

A limitation of the current study is the small number of horses included in the in vivo component. This limits the ability to evaluate the potential effects of factors such as sex, breed, and physiological status on drug metabolism. In addition, liver microsomes were obtained from a single horse; therefore, all enzymatic kinetic parameters reported here are based on a single animal. It is also important to note that although a large number of recombinant enzymes were assessed, it is possible that undiscovered CYP enzymes may contribute to vonoprazan biotransformation.

Another limitation of the present study is that metabolite evaluation was restricted to plasma samples, and urinary excretion was not assessed. Evaluation of plasma samples at a single timepoint, did not indicate the presence of glucuronidated metabolites; however, this does not completely exclude their formation and subsequent elimination via the urine. It is possible that glucuronide conjugates of vonoprazan and/or its metabolites may be present in urine but were not detected in the circulation, as they may be present at concentrations below the detection limit of the analytical assay. In dogs, the primary route of excretion for vonoprazan and the metabolites M-I and M-II is urine, whereas in rat fecal excretion predominates [14]. Analysis of urine would also be valuable to further characterize the elimination pathways of vonoprazan in horses, including the potential contribution of renal excretion of the parent compound [10]. Finally, the present work focused exclusively on the metabolism of vonoprazan to the M-I and M-III metabolite, as these were the only two metabolites in which analytical standards were commercially available. It is important to note that other metabolites, such as M-II and vonoprazan N-sulfate have been identified in other species and may also be generated by horses.

## 5. Conclusions

Vonoprazan administration to horses resulted in the formation of the metabolites M-I and M-III, consistent with reports in other species. Although vonoprazan metabolites have been described as pharmacologically inactive in humans, their pharmacological activity in horses has not yet been established. Of the CYP450 enzymes studied, CYP2D50 and CYP3A94 appeared to be the primary enzymes involved in vonoprazan metabolism in horses. Additional studies are warranted to further characterize vonoprazan metabolism in horses, including urinary elimination, generation of additional metabolites, and the potential for drug–drug interactions.

## Figures and Tables

**Table 1:** Cytochrome P450 (CYP450) enzymes used and current accession numbers.

CYP450 Family	Enzyme Name	Accession Number
CYP1A	CYP1A1	XM_023653657.1
	CYP1A2	XM_001493886.5
CYP2B	CYP2B6	NM_001317266.1
CYP2C	CYP2C19	XM_001502179.5
	CYP2C21	XM_001501993.4
	CYP2C23	XM_014733407.1
	CYP2C42-1	XM_001502162.3
	CYP2C42-3	XM_001500745.2
	CYP2C92	NM_001101652.1
	CYP2C113	NM_001291302.1
CYP2D	CYP2D50	NM_001111306.1
	CYP2D82	XM_023631038.1
CYP3A	CYP3A89	NM_001101651.1
	CYP3A93	NM_001190938.1
	CYP3A94	NM_001190939.1
	CYP3A95	NM_001190940.1
	CYP3A96	NM_001146163.2
	CYP3A97	NM_001146164.1
CYP4A	CYP4A11	XM_023630431.1

**Table 2:** Pharmacokinetic parameters (mean ± standard deviation; T_max_, median and range) for MI (metabolite I; vonoprazan-oxide) and MIII (metabolite III; vonoprazan-nitrone) following oral administration of 0.5 and 1 mg/kg vonoprazan to six horses.

	Vonoprazan Dose: 0.5 mg/kg		Vonoprazan Dose: 1 mg/kg	
Parameter	MI	MIII	MI	MIII
C_max_ (ng/mL)	11.9 ± 8.39	0.377 ± 0.238	39.2 ± 28.3	1.67 ± 1.66
C_max_/Dose (ng/mL)	23.8 ± 16.78	0.75 ± 0.476	39.2 ± 28.3	1.67 ± 1.66
T_max_ (h)	1 (1–2)	1 (1–8)	1.5 (1–8)	1 (1–8)
AUC_0__→__inf_ (h*ng/mL)	60.0 ± 25.08	2.95 ± 3.06	180.1 ± 86.44	6.60 ± 4.49
%AUC_extrap_	5.62 ± 3.62	38.3 ± 25.8	1.33 ± 0.98	17.9 ± 20.0
λ_z_ (1/h)	0.064 ± 0.02	0.187 ± 0.13	0.101 ± 0.03	0.257 ± 0.11
HL λ_z_ (h)	11.6 ± 1.58	5.23 ± 2.94	7.07 ± 1.35	3.09 ± 1.97

C_max_: maximum drug concentration; C_max_/Dose: maximun drug concentration corrected for dose; T_max_: time of maximal concentration; AUC_0 →inf_: area under the curve extrapolated to infinity; %AUC_extrap_: percentage of the area under the curve extrapolated; **λ_z_**: slope of the terminal portion of the concentration curve; HL λ_z_: half-life lambda z.

**Table 3:** Estimates of in vitro kinetic parameters for vonoprazan-N-oxide (M-I) and vonoprazan nitrone (M-III) formation following incubation with equine liver microsomes (ELMs) and equine recombinant cytochrome P450 enzymes (eq-rCYP2D50 and eq-rCYP3A94).

System	Metabolite	Model (Range)	V_max_ (pmol/min/pmol CYP450)	K_m/_K_half_ (µM)	K_i_ (µM)	Hill (h)	Apparent CL_int_
ELM	M-I	Substrate inhibition (0–800 µM)	0.01096	16.54	2105	—	6.63 × 10^−4^
	M-III	Michaelis–Menten (0–800 µM)	0.001361	2.412	—	—	5.64 × 10^−4^
eq-rCYP2D50	M-I	Substrate inhibition (0–1000 µM)	0.002937	1.476	1097	—	1.99 × 10^−3^
	M-III	Linear (0–400 µM)	—	—	—	—	—
eq-rCYP3A94	M-I	Michaelis–Menten (0–400 µM)	0.0007194	4.045	—	—	1.78 × 10^−4^
	M-III	Allosteric sigmoidal (0–400 µM)	0.006399	13.23	—	1.824	—

Apparent intrinsic clearance was calculated as V_max_/K_m_ for saturable systems. —: not applicable; CL*_int_:* intrinsic clearance, enzyme capacity to clear to substrate without protein-binding or organ blood flow limitations; K_m_ (for Michaelis–Menten) and K_half_ (for allosteric sigmoidal): concentration of substrate that produces a half-maximum enzyme velocity; h: hill of the slope (h > 1 indicates sigmoidal curve due to positive cooperativity); Ki: substrate inhibition constant.

## Data Availability

Dataset available on request from the authors.

## Abbreviations

AUC_0__→inf_	Area under the curve extrapolated to infinity
Clint	Intrinsic clearance
Cmax	Maximum drug concentration
Cmax/Dose	Maximun drug concentration corrected for dose
CYP	Cytochrome enzyme
EGUS	Equine gastric ulcer syndrome
ELM	Equine liver microsome
Eq-r	Equine recombinant
HL λz	Half-life lambda z
h	Hill of the slope
Ki	Substrate inhibition constant
M-I	Metabolite I
M-II	Metabolite III
P-CAB	Vonoprazan is a potassium-competitive acid blocker
%AUCextrap	Percentage of the area under the curve extrapolated
Tmax	Time of maximal concentration
λz	Slope of the terminal portion of the concentration curve
